# BeiDou Augmented Navigation from Low Earth Orbit Satellites

**DOI:** 10.3390/s19010198

**Published:** 2019-01-07

**Authors:** Mudan Su, Xing Su, Qile Zhao, Jingnan Liu

**Affiliations:** 1 GNSS Research Center, Wuhan University, 129 Luoyu Road, Wuhan 430079, China; PeonySu@whu.edu.cn (M.S.); jnliu@whu.edu.cn (J.L.); 2 College of Geomatics, Shandong University of Science and Technology, No. 579 Qianwangang Road, Qingdao 266590, China; 3 Beijing Institute of Tracking and Telecommunications Technology, No. 26 Beiqing Road, Beijing 100094, China; 4 Collaborative Innovation Center of Geospatial Technology, Wuhan University, 129 Luoyu Road, Wuhan 430079, China

**Keywords:** navigation, LEO, PPP, ambiguity convergence time

## Abstract

Currently, the Global Navigation Satellite System (GNSS) mainly uses the satellites in Medium Earth Orbit (MEO) to provide position, navigation, and timing (PNT) service. The weak navigation signals limit its usage in deep attenuation environments, and make it easy to interference and counterfeit by jammers or spoofers. Moreover, being far away to the Earth results in relatively slow motion of the satellites in the sky and geometric change, making long time needed for achieved centimeter positioning accuracy. By using the satellites in Lower Earth Orbit (LEO) as the navigation satellites, these disadvantages can be addressed. In this contribution, the advantages of navigation from LEO constellation has been investigated and analyzed theoretically. The space segment of global Chinese BeiDou Navigation Satellite System consisting of three GEO, three IGSO, and 24 MEO satellites has been simulated with a LEO constellation with 120 satellites in 10 orbit planes with inclination of 55 degrees in a nearly circular orbit (eccentricity about 0.000001) at an approximate altitude of 975 km. With simulated data, the performance of LEO constellation to augment the global Chinese BeiDou Navigation Satellite System (BeiDou-3) has been assessed, as one of the example to show the promising of using LEO as navigation system. The results demonstrate that the satellite visibility and position dilution of precision have been significantly improved, particularly in mid-latitude region of Asia-Pacific region, once the LEO data were combined with BeiDou-3 for navigation. Most importantly, the convergence time for Precise Point Positioning (PPP) can be shorted from about 30 min to 1 min, which is essential and promising for real-time PPP application. Considering there are a plenty of commercial LEO communication constellation with hundreds or thousands of satellites, navigation from LEO will be an economic and promising way to change the heavily relay on GNSS systems.

## 1. Introduction

The Global Navigation Satellite System (GNSS) represents a constellation of satellites providing signals from space, transmitting positioning and timing data with global coverage [1]. A GNSS receiver employs trilateration to determine its position on or near the Earth’s surface by timing signals from four or more GNSS satellites. Currently, GNSS is well recognized as the major enabler of ‘precision’, and widely used for the precise timing and positioning. There are two fully operational GNSS systems at present: the United States’ Global Positioning System (GPS), and the Russian Federation’s Global Navigation Satellite System (GLONASS). Two systems under development, the Chinese Beidou Navigation Satellite System (BeiDou), and the European Union’s Galileo system, which are both expected to achieve full global coverage capability by 2020. In addition, the Japanese Quasi-Zenith Satellite System (QZSS) and Indian Navigation with Indian Constellation (NAVIC) are two regional systems to provide the augmentation service. The Multi-GNSS constellations provide the orbit as well as frequency diversity to strengthen the observation geometry and reduce the transmitting errors, resulting in improvement of positioning accuracy and stability [2].

The above-mentioned GNSS systems are consisted of the satellites at Geostationary orbit (GEO), Inclined Geostationary orbit (IGSO) or Medium Earth Orbit (MEO), and all of them are 20×~30× further from the Earth compared to the LEO. Hence, the energy of navigation signals is significantly spread out during their passage to the Earth, resulting in too weak signals to be used reliably in deep attenuation environments such as indoors or in city canyons. Moreover, the GNSS signals are susceptible to interference and counterfeit by jammers or spoofers [3]. From the view of high accuracy positioning with carrier-phase measurements, being far away to the Earth results in relative slow motion of the satellites in the sky and geometric change with respect to the ground stations. Hence, a quite long time (normally up to more than 30 min) is needed for the ambiguities to be reliably converged to the correct integer values [2]. By using the satellites at Lower Earth Orbiter (LEO) as the navigation satellites, these disadvantages can be improved [4,5,6].

LEO satellites (with altitude from 160 km to 2000 km) are 20 times closer to the Earth than GNSS at MEO, leading to stronger radio signals on the ground for navigation because of the lower path loss of the signal. The swift motion of LEO provides the rapid geometric change for rapid integer ambiguity resolution. The other advantages of LEO used as navigation satellites include whitening multipath and effective Doppler positioning, as the rapid motion of LEO makes the reflected signals are no longer effectively static over short averaging times and strengthens the Doppler shift [7]. However, being them too close to the Earth, the ground converge of LEO satellite is less than one-tenth of that of MEO. Hence, the hundreds of LEO satellites are needed to match the coverage of GNSS [3]. It is extremely expensive to deploy such a massive number of LEO satellites to only provide navigation service. Thanks to the development of commercial communication LEO constellation, it is promising and possible to integrate the navigation components to the communication satellites. The summary of some deployed or proposed commercial communication LEO constellations is given in Table 1.

The Iridium satellite constellation was proposed in 1987 and became operational in 1998 to provide voice and data coverage to satellite phones. The constellation consists of 66 active satellites and additional backup satellites. The operational satellites are deployed in six orbital planes spaced 30° apart, with 11 satellites in each plane. The orbit altitude and inclination are approximately 781 km and 86.4°, respectively [8]. The second generation Iridium-NEXT satellites began to be deployed into the existing constellation in January 2017. Somewhat similar to the Iridium satellite constellation, Globalstar is also a LEO satellite constellation for satellite phone and low-speed data communications, the first generation Globalstar constellation have 48 satellites with an orbital height of approximately 1400 km and an inclination of 52°. A Globalstar second-generation constellation consists of 24 LEO satellites, which were fully launched in 2013.

To keep up with the rising demand for broadband, several new LEO constellations are being proposed. Proposed in 2013, LeoSat is designed to consist of 108 satellites in 1400 km orbit and use Ka-band for provide secure broadband links [9]. The Canadian Telesat proposed a LEO communication system consists of a constellation of 117 satellites in 11 orbital planes, with six planes (12 satellites per plane) inclined 99.5 degrees in a circular orbit at an approximate altitude of 1000 km and five planes (nine satellites per plane) inclined 37.4 degrees in a circular orbit at an approximate altitude of 1248 km [10]. In January 2015, OneWeb announced a partnership with Virgin and Qualcomm to produce a satellite internet constellation of approximately 720 satellites expected to provide global Internet broadband service to individual consumers as early as 2019. These satellites evenly distributed in 18 near-polar orbital planes, at an approximate altitude of 1200 km. Moreover, the system will be expended to accommodate additional 1260, which would double the number of orbital planes from 18 to 36, and increase the maximum number of satellites per plane from 40 to 55 [11]. SpaceX, with support from Google, announced a similar ambition for StarLink constellation with 4425 LEO satellites operating in 83 orbital planes at altitudes ranging from 1100 km to 1325 km in March 2018, and this new approval gives the green light for the planned 7518 satellites in the Starlink constellation [12,13]. The prototype test-flight satellites were successfully launched on 22 February 2018. In August of 2015, Samsung expressed interest with a proposal for a LEO constellation of 4600 [14]. Boeing joined the race in June of 2016 announcing plans for a LEO constellation of 2956 satellites to provide broadband internet access globally. Boeing plans to deploy the first portion of the system, 1396 satellites operating at an altitude of 1200 km, and to subsequently increase the constellation to the total of 2956 satellites [15]. Moreover, China Aerospace Science and Industry Corporation (CASIC) have launched a project named ‘Hongyun’ to build a global wideband communications LEO satellite constellation consisting of 156 satellites with altitude 1000 km. The first phase of the project is expected to complete by 2021, and the first experimental satellite has been launched in 2017 [16]. Meanwhile, in late-February 2018, the China Aerospace Science and Technology Corporation (CASC) announced plans to build the ‘Hongyan’ LEO constellation of 324 small satellites for global communications and other services. The Hongyan constellation with 1100 km altitude is targeted to be operational by 2021 [17].

Currently, the Iridium constellation is already broadcasting structured independent navigation signals, and provides independent positioning, navigation and timing (PNT) service based on Satelles’ satellite time and location (STL) signal technology to augment or serve as a back-up to existing GNSS PNT solutions [18]. STL is designed such that a receiver can reliably decode the bursts and perform precise Doppler and range measurements at attenuation of up to 39 dB relative to unobstructed reception. This is sufficient to penetrate buildings and other occlusions, providing coverage in most deep indoor and urban canyon environments. The testing results show that STL always experienced strong signals and can provide sub-microsecond timekeeping even in deep attenuation environments such as indoors. In addition, after 10 min of convergence, the STL solution had converged to an accuracy of better than 35 m with same accuracy in vertical and horizontal direction [19].

Used as the independent navigation satellite system, a PNT service comparable to GPS could be achieved although with the added benefits of LEO, including stronger signals and rapid changes in geometry. Besides, the LEO can also be used to augment the GNSS to provide combined solution with GNSS and LEO navigation signals. The aim of this study is to assess the possibility and performance of LEO constellation used to augment the BeiDou navigation system for precise positioning. In Section 2, the advantage of LEO navigation system in positioning accuracy and integer ambiguity resolution will be analyzed theoretically. In Section 3, the strategy used for simulating the observations from GNSS and LEO constellation as well as Precise Point Positioning (PPP) with GPS-only or combined GNSS and LEO will be presented. The results will be analyzed and discussed in Section 4 with emphasizing on the convergence of ambiguity as well as accuracy after convergence. Finally, the study will be concluded in Section 5.

## 2. Advantages of Navigation with LEO Constellation

### 2.1. Positioning Error

Like GNSS systems, the users will also use the trilateration to compute their position with navigation service provided by LEO constellation. Hence, statistically, the point positioning accuracy can be described by the product of the user equivalent range error (UERE), which combines all measurement and modeling errors of an individual pseudo-range, and the dilution of precision (DOP), which maps such errors to the position uncertainty for a given number and geometric distribution of observed navigation satellites [20]. The relation can be expressed as following.

(1) $$\sigma_{3D}=PDOP\cdot \sigma_{UERE}$$

where $\sigma_{3D}$ is the user 3D root-mean-square (RMS) positioning error, PDOP is the position DOP, and $\sigma_{UERE}$ is the UERE.

The DOP factors are derived from the terms associated with the user satellite geometry. Therefore, only geometry maps range errors into user space. This means that DOP is the geometry factor of the visual satellites related to their distribution. Whether the satellites are distributed more homogeneously, it will be helpful to the position estimation for the observation information gathered from more directions. Usually, the DOP value is resolved into horizontal (HDOP) and vertical dilution of precision (VDOP), these give us the relationship between UERE and errors in the vertical and horizontal directions. Thanks to the fast motion and numerous satellites, the DOP will be significantly improved by LEO constellation. The simulated results will be presented in Section 4.1.

The UERE is further divided into a signal-in-space [user] range error (SIS URE or SISRE) and a user equipment error (UEE). While the UEE term comprises all receiver-related contributions such as noise, multipath and uncorrected atmospheric errors, the SISRE describes the statistical uncertainty of the modeled pseudo-range due to errors in the broadcast orbit and clock information. It is driven by the space segment characteristics (e.g., clock stability and predictability of orbital motion), as well as the control segment capabilities (orbit and clock determination performance, distribution of monitoring stations and upload capacity). The orientation of the line-of-sight vector impacts the orbit errors $\Delta r=\left(\Delta r_{R},\Delta r_{A},\Delta r_{C}\right)$ in the radial ($\Delta r_{R}$), along-track ($\Delta r_{A}$) and cross-track ($\Delta r_{C}$) directions on the line-of-sight range, and hence the user location. The average contribution over all points of the Earth within the visibility cone of the satellite is considered normally for a statistical description. The orbit-only contribution to the SISRE can then be described as a weighted average:

(2) $$SISRE\left(orb\right)=RMS\left[w_{R}^{2}\cdot \Delta r_{R}^{2}+w_{A,C}^{2}\cdot \left(\Delta r_{A}^{2}+\Delta r_{C}^{2}\right)\right]=\sqrt{w_{R}^{2}\cdot R^{2}+w_{A,C}^{2}\cdot \left(A^{2}+C^{2}\right)}$$

of the RMS errors $R=RMS\left(\Delta r_{R}\right)$, $A=RMS\left(\Delta r_{A}\right)$ and $C=RMS\left(\Delta r_{C}\right)$ scale like text along the three axes. The altitude of the GNSS satellite determines the weight factors $w_{R}$ and $w_{A,C}$ [21]. For MEO, the $w_{R}$ is closed to 1, and it indicates that the radial orbits largely project onto the user. However, for LEO orbits, the weight factors become almost equally important to the SISRE [3], as their cones should be wider to provide larger converge. The combined orbit and clock SISRE is obtained from the expression:

(3) $$SISRE=\sqrt{{\left(w_{R}\cdot \Delta r_{R}-\Delta cdt\right)}^{2}+w_{A,C}\cdot \left(A^{2}+C^{2}\right)}$$

the Δcdt represents the error of the broadcast clock offset. It takes into consideration that the modeled pseudo-range is derived from the difference of the radial orbit error and the clock offset. The analysis performed by Montenbruck et al. [21] indicates that the SISRE for GPS, GLONASS, Galileo, QZSS, and BeiDou regional system (BeiDou-2) are 0.71 m, 1.97 m, 1.64 m, 0.57 m, and 1.46 m, respectively. For the UEE term, although it varies with different GNSS system and correction model used, the uncertainty is about 1.2 m, 0.2 m, and 1.0 m for ionosphere and troposphere modeling erros as well as Equipment Receiver Noise & Multipath. In total, the UERE is about 1.77 m, 2.51 m, 2.01 m, 1.66 m, and 1.96 m for GPS, GLONASS, Galileo, QZSS, and BeiDou-2. For the LEO navigation constellation, as the same errors are exist in receiver side as well as when the signals pass through the atmosphere, the UEE for LEO constellation may have similar magnitude as that of GNSS. Hence, the clock and orbit accuracy of LEO constellation are essential for SISRE of the LEO navigation constellation. For LEO navigation constellation, three potential data can be used for orbit and clock determination, e.g., ground tracking data, intersat link data, and onboard GNSS tracking data. With ground tracking or intersat link data, as GNSS satellites, the accuracy of orbit and clock are rely on the predicted one. However, as closer to the satellites, the perturbation acting on the LEO are hard to model, resulting in poor prediction ability for longer orbit. Hence, the frequency updating of the broadcast ephemerics of LEO is needed. As to the analysis by Reid [22], the 3D RMS of orbit error can reach to about 1.5 m. Once the onboard GNSS data available, the real-time onboard solution can be obtained with accuracy about 1.5 m in 3D. For the clock, the worst uncertainty is about 6.0 ns (approximate 1.8 m), that because the LEO use the chip-scale atomic clocks (CSAC) for the restriction of power consumption, size, weight, and cost etc.

### 2.2. Integer Ambiguity Resolution

For high accuracy positioning using the carrier-phase measurements (e.g., PPP), the positioning accuracy is rely on if the ambiguities can be correctly fixed to be integer. Hence, the convergence speed of ambiguity solution has essential impact on the positioning solution. Normally ambiguities are solved at the same time with the other parameters such as station coordinates, receiver clocks, troposphereic delay, and so on. With the change of observation geometry, the multipath and atmospheric delay is estimated quickly, making that the ambiguities can be separated with those parameters. In this case, a reliable ambiguity solution can be obtained. The probability of ambiguity solution is dependent on the satellite types and altitude and the empirical formula can be expressed as [23]:

(4) $$P\left(ambiguity\right)=f\left(\dot{s},PDOP\right)=\kappa \frac{\dot{s}}{PDOP}$$

where κ coefficient related to frequency, atmosphere and so on; P probability; $\dot{s}$ change rate of line-of-sight between tracking station and satellite. The ambiguity solution related to types of satellite orbits using GPS L1 frequency as an example is approximately shown in Table 2.

The results demonstrated that the probability of ambiguity solution is dependent on the PDOP and the change rate of line-of-sight between tracking station and satellite. For the satellite at lower orbit, the swift motion makes that the rate of line-of-sight between tracking station and satellite change rapidly, and improve the geometry and PDOP. In this case, the ambiguity can be converged to the integer in quite short period. However, for higher altitude, longer time is needed for integer ambiguity resolution. In particular, for GEO, the ambiguity cannot be solved if there are no multi-frequency observations.

## 3. Strategy for Data Simulation and Procession

**Figure 1:**
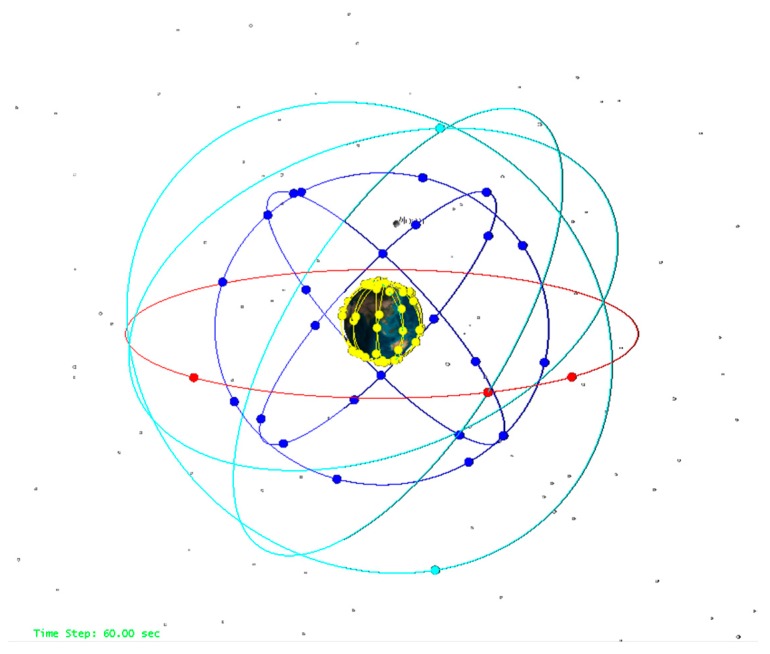
The satellite distribution of the BeiDou-3 and LEO constellation.

In order to assess the independent and integrated navigation performance of LEO constellation, the ground observations for BeiDou global (BeiDou-3) constellation and a LEO constellation were simulated. According to the Interface Control Document [24], the BeiDou-3 constellation will consist of three GEO, three IGSO, and 24 MEO satellites. The three GEO satellites will operate in the orbit at an altitude of 35,786 km, and are located at 80°E, 110.5°E, and 140°E, respectively. The IGSO satellites with same altitude as GEO have an inclination of the orbital planes of 55 degrees. The MEO satellites will operate in orbit with altitude of 21,528 km and inclination of 55 degrees [24]. The simulated LEO navigation system consists of a constellation of 120 satellites in 10 orbit planes with inclination of 55 degree in a nearly circular orbit (eccentricity about 0.000001) at an approximate altitude of 975 km. As the main aim of the proposed LEO constellation is to argument the BeiDou-3 system, in particularly providing the high accuracy positioning in China. With the initial orbit elements, the 24 h orbits for these BeiDou-3 and LEO satellites can be generated with numerical integration based on the force models summarized on Table 3. Figure 1 shows the distributions of BeiDou-3 and LEO constellation in the space.

**Figure 2:**
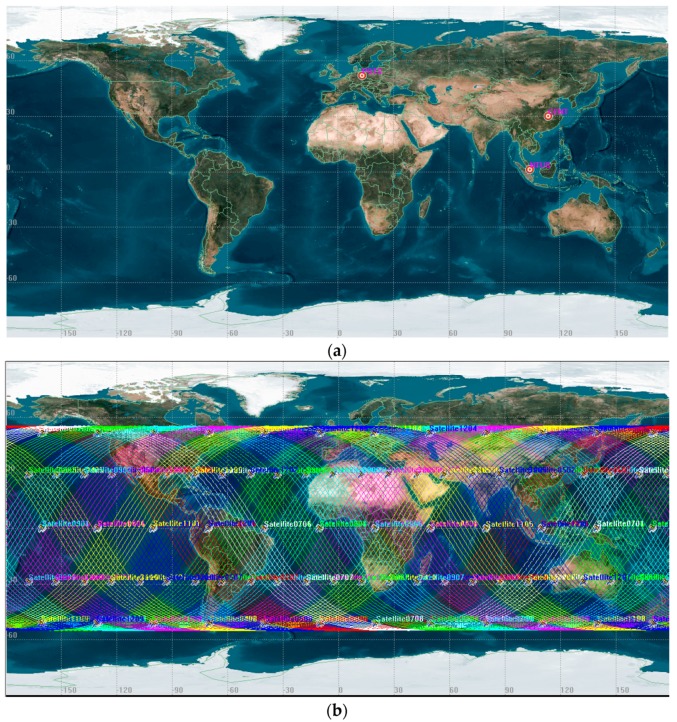
The distribution of selected ground stations (**a**) and ground track of LEO constellation (**b**).

Three ground stations (CENT located in Wuhan, latitude 30.52779, longitude 114.35686; POTS located in Potsdam, latitude 52.37929, longitude 13.06609; NTUS located in Singapore, latitude 1.34580, longitude 103.67995) are selected for simulating the ground tracking data to demonstrate the positioning performance of the BeiDou-3 and LEO constellation in different regions. Figure 2 shows the distribution. The observations are simulated based on the following observation equations of code and phase:

$$\begin{array}{l} \rho_{r,i}^{s}=R_{r}^{s}+cdt_{r}-cdt^{s}+m_{r}^{s}T_{r}+\frac{f_{i}^{2}}{f_{1}^{2}}I_{r}^{s}+b_{r,i}-b_{i}^{S}+\delta_{PCO,i}+\delta_{PCV,i}+\delta_{rel}+\delta_{mul,\rho_{r,i}^{s}}+\varepsilon_{\rho_{r,i}^{s}}\\ \varphi_{r,i}^{s}=R_{r}^{s}+cdt_{r}-cdt^{s}+m_{r}^{s}T_{r}-\frac{f_{i}^{2}}{f_{1}^{2}}I_{r}^{s}+\lambda_{i}N_{r}^{s}+B_{r,i}-B_{i}^{S}+\delta_{PCO,i}+\delta_{PCV,i}+\delta_{rel}+\delta_{mul,\varphi_{r,i}^{s}}+\delta_{wind-up}+\varepsilon_{\varphi_{r,i}^{s}} \end{array}$$

where the subscript i denote the number of frequency with frequency value $f_{i}$. The subscript r and the superscript s denote receiver and satellite, respectively. $\rho_{r,i}^{s}$ and $\varphi_{r,i}^{s}$ are code and phase range at frequency i which need to be simulated. $R_{r}^{s}$ denotes satellite-to-receiver distance. $dt_{r}$ and $dt^{s}$ denote receiver and satellite clock. $T_{r}$ and $I_{r}^{s}$ denote tropospheric and ionospheric delay. $b_{r,i}$ and $b_{i}^{S}$ indicate the biases for code in the receiver and satellites sides at frequency i, while $B_{r,i}$ and $B_{i}^{S}$ are the corresponding biases for phase. These biases terms are omitted in the simulation, resulting in no biases between different observations and system. The term $\lambda_{i}$ denotes wavelength and $N_{r}^{s}$ means integer ambiguity. $\delta_{PCO,i}$ and $\delta_{PCV,i}$ are the phase centre offset (PCO) and phase centre variation (PCV) at frequency i, respectively. $\delta_{rel}$ and $\delta_{wind-up}$ are the relative effect and phase wind-up. $\delta_{mul,\rho_{r,i}^{s}}$ and $\delta_{mul,\varphi_{r,i}^{s}}$ are the multipath error for code and phase measurement. The terms $\varepsilon_{\rho_{r,i}^{s}}$ and $\varepsilon_{\varphi_{r,i}^{s}}$ denote the code and phase observation errors, respectively.

The simulation is mainly to calculate all the components on the right side of observation equations. The satellite-to-receiver distance is computed with the receiver and satellite positions. Specifically, this distance is associated with the mass centers of the satellite and the receiver. Thus, this distance needed to corrected by the PCO and PCV of receiver and satellite. The receiver clock error of the ground tracking stations are with uncertainty about 10 us (3000 m), while the satellite clock are interpolated from the precise clock error file. The ionospheric delay is ignored as we use the ionosphere-free combination observations, and the second and third ionospheric delays are too small to consider in the data processing. For the tropospheric delay, we use the Saastamoinen model [27] and global mapping functions (GMF) [28] for the ground tracking network. Moreover, relativistic correction, phase windup correction, and tidal displacement are all taken into consideration. The ambiguities were set as zero. For GNSS satellites, the nominal yaw-steering attitude mode is used [21], which the LEO’s attitude is assumed along the along-track, cross-track, and radial direction. The noises for code and phase are set as 1 m and 2 mm. Both of the LEO and GNSS satellites used the same frequency for the simulation. Table 4 summarizes the configuration.

Once the observations are generated, PPP are performed for the station to investigate the accuracy and convergence time achieved by BeiDou-3 only and BeiDou-3/LEO combined solutions. In this case, the epoch-wise station coordinates, epoch-wide receiver clock, hourly troposphere zenith delays, and ambiguity are estimated. As no inter-system bias was simulated, this term were not be estimated when BeiDou-3 is combined with LEO for PPP [29]. The ionosphere-free linear combination was used, with 2 mm observational standard deviation applied in the weighting of both the BeiDou-3 and LEO phase observations, and 1 m for both the BeiDou-3 and LEO code observations. Once the 3D positioning accuracy within ten continuous epochs is less than 10 cm, the positioning is treated as convergence.

## 4. Results and Discussion

In this section, the performance of LEO constellation used to argument the BeiDou-3 is assessed with several metrics including satellite visibility, PDOP, accuracy and convergence of positioning.

### 4.1. Satellite Visibility

**Figure 3:**
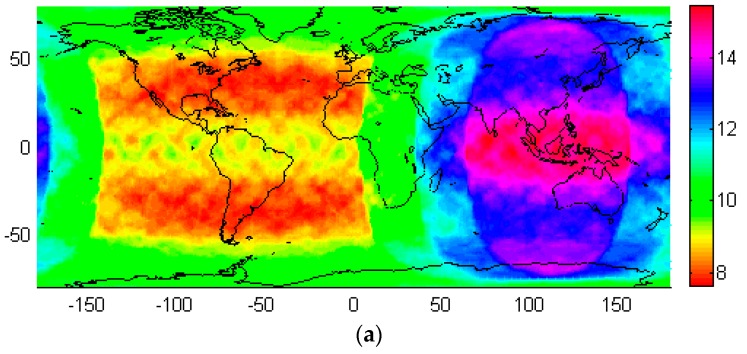

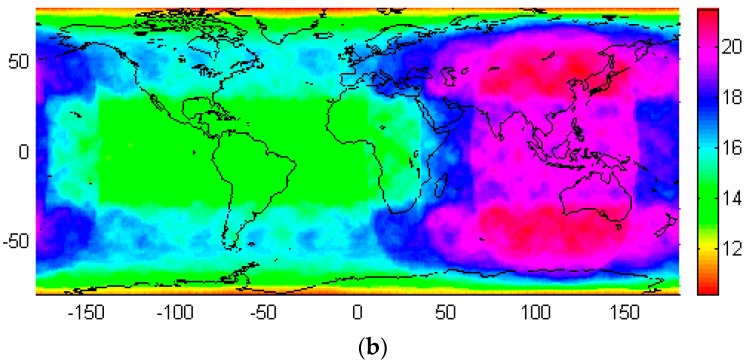
Global distribution for satellite visibility of BeiDou-3-only (**a**) and BeiDou-3/LEO combined constellation (**b**).

The satellite visibility of BeiDou-3 only as well as BeiDou-3 and LEO constellation is computed. With dividing the global to the grids with 5° in latitude and 5° longitude, the number of satellites tracked by the location in the center of grid is calculated with 0 mask elevation and 30 s data sampling interval. Afterword, the average number for 1 day is used as the indicator of satellite visibility. Figure 3 demonstrates the global distribution of the satellite visibility for BeiDou-3 only (Figure 3a) and BeiDou-3 as well as LEO constellation (Figure 3b). For BeiDou-3, in general, the satellite visibility is distributed symmetrically in Northern hemisphere and Southern hemispheres, but unevenly in the Eastern hemisphere and Western hemisphere. More than 12 satellites can be tracked in Asia-Pacific region, where the 3 GEO and 3 IGSO satellites are located. In particular, for the low latitude region in the Asia-Pacific region, up to 15 satellites can be tracked. However, the number of tracked satellites are lower in the western hemisphere, particularly for the mid-latitude region of north and South American, there are only 8 satellite can be tracked in averaged. For the region with latitude beyond 55°, about 10 satellites are in view, as the orbital inclination of BeiDou-3 MEO satellites are set as 55°.

Once the 120 LEO satellites have been incorporated, although the global distribution is still uneven in Western and Eastern hemisphere, the satellite visibility has been improved significantly, particularly in the mid-latitude region, as the low-orbit navigation satellites are designed to have an orbital inclination of 55°. In China, at least 18 satellites can be tracked, particularly it can be reach to more than 20 in the north part of China. The great satellite visibility can improve the accuracy obviously, and explain the reason for selection of such kind of LEO constellation configuration. Compared to that of BeiDou-3 only, the satellite visibility in the mid-latitude region of north and South American has been improved, and more then 14 satellites can be tracked. However, the contribution of LEO to the satellite visibility is limited, as the limitation of orbital inclination as well as ground coverage of LEO satellites. Table 5 lists the statistical results for the two configurations. In general, once the LEO satellites have been incorporated, the maximum number of visible satellites has increased from 15.5 to 21.6. Meanwhile, the minimum number of visible satellites has increased from 7.6 to 10.1. Most significant improvement can be observed by the averaged visible satellite, which is increased from 10.7 to 16.3, indicating that the LEO has greatly improved the performance of navigation services worldwide.

### 4.2. PDOP

**Figure 4:**
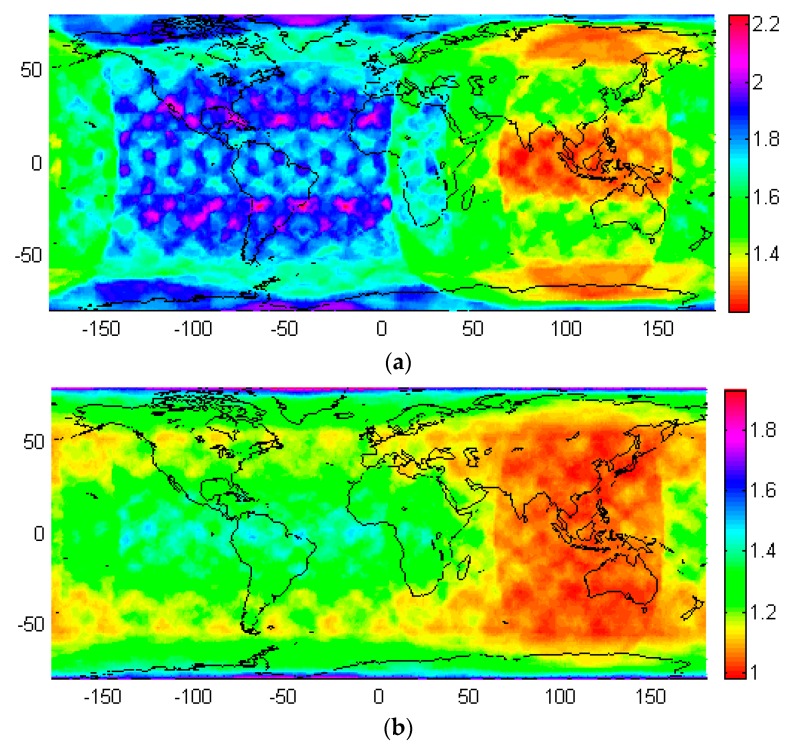
Global distribution for PDOP of BeiDou-3-only (**a**) and BeiDou-3/LEO combined constellation (**b**).

The PDOP indicates the positioning accuracy theoretically, and is related to the number of satellite tracked as well as the distribution of satellites. In general, the smaller the PDOP value is, the better accuracy for positioning is. Similar as calculation of satellite visibility, the averaged PDOP with 24 h data for the location in the center of each 5° × 5° grid is obtained with with 0 mask elevation and 30 s data sampling interval. Figure 4 demonstrates the global distribution of the PDOP for BeiDou-3 only and BeiDou-3 (Figure 4a) as well as LEO constellation (Figure 4b). Similar as satellite visibility, the PDOP is distributed symmetrically in Northern hemisphere and Southern hemispheres, but unevenly in the Eastern hemisphere and Western hemisphere. For BeiDou-3, the PDOP value reaches a minimum of 1.2–1.3 in the low latitudes of Asia-Pacific region, where is the great tracking ability for the three GEO and three IGSO satellites. In most areas of China, the PDOP value is about 1.5. As less satellites can be tracked, the mid-latitude regions of the north and south Americas have the largest PDOP up to 2.2. The poor positioning ability (with PDOP up to 2.2) in the north and south pole region is related to the configuration of BeiDou-3 satellites, as above mentioned, the 55° orbit inclination of MEO satellites limits the satellite visibility in these regions, resulting in larger PDOP.

Once the LEO satellites have been used to augment the BeiDou-3, the navigation service performance in mid-latitude regions has been significantly improved. The PDOP values in the Asia-Pacific region were around 1, and low-latitude and mid-latitude show similar performance. For PDOP in the mid-latitude region of north and south Americas, it has been reduced to approximate 1.2 from abound 1.8. Thanks to the 55° orbital inclination used for LEO satellites, the PDOP has been improved to about 1.2 in the region with latitude about 55° and −55°. However, limited improvement is identified in low latitude region of north and south Americas as well as the pole regions. In statistics, as listed in Table 6, the maximum PDOP value was reduced from 2.24 to 1.93, the minimum PDOP value was decreased from 1.19 to 1.01, and the average PDOP value was reduced from 1.63 to 1.22. This again confirms that the LEO constellation can greatly improve the performance of BeiDou-3.

### 4.3. SPP

The standard point positioning (SPP) with pseudo-range measurements was carried to investigate the performance of incorporating LEO constellation to augment BeiDou-3 for the selected station. The simulated code measurements with 1 m random noise were processed with precise satellites’ orbit and clock for observation simulation. The ionosphere delay has been corrected with ionosphere-free combination, and the wet and dry troposphere delay was corrected with Saastamoinen model and global mapping functions (GMF). In this case, only four epoch-wise parameters including coordinates and receiver clock were estimated. Two solutions with BeiDou-3 only or combined BeiDou-3 and LEO measurements were performed.

In general, the inclusion of LEO data has limited effect on SPP. Taking CENT station for example, the positioning accuracy has been improved from 105.3 to 100.7 cm in earth direction, 99.1 to 93.2 in north direction, as well as 293.7 to 261.3 cm in Up direction, once LEO data have been used. Table 7 shows the SPP precision of these three stations in north, east and up direction.

### 4.4. PPP

In order to investigate the contribution of LEO to accuracy and convergence of high-accuracy positioning with carrier-phase measurements, two PPP experiments have been performed with BeiDou-3 or combined BeiDou-3 and LEO measurements. The simulated code and phase measurements with 1 m and 2 mm random noise were processed with precise satellites’ orbit and clock. The ionosphere delay has been corrected with ionosphere-free combination, and the dry troposphere delay was corrected with the Saastamoinen model and global mapping functions (GMF), and the wet delay has been estimated per hour. Besides, coordinates and receiver clock, the constant and inter-system bias were estimated.

**Figure 5:**
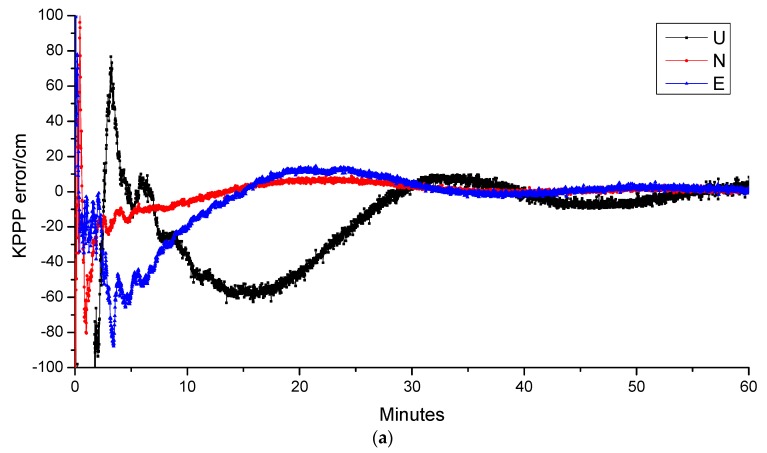

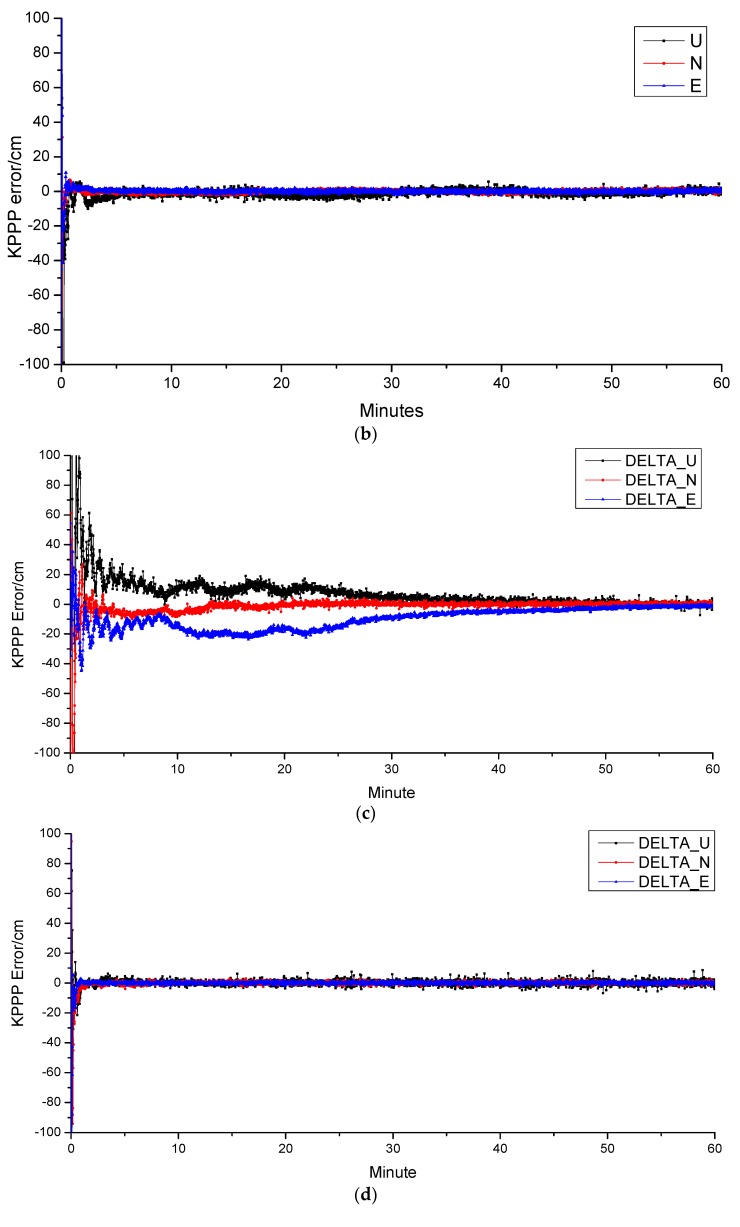

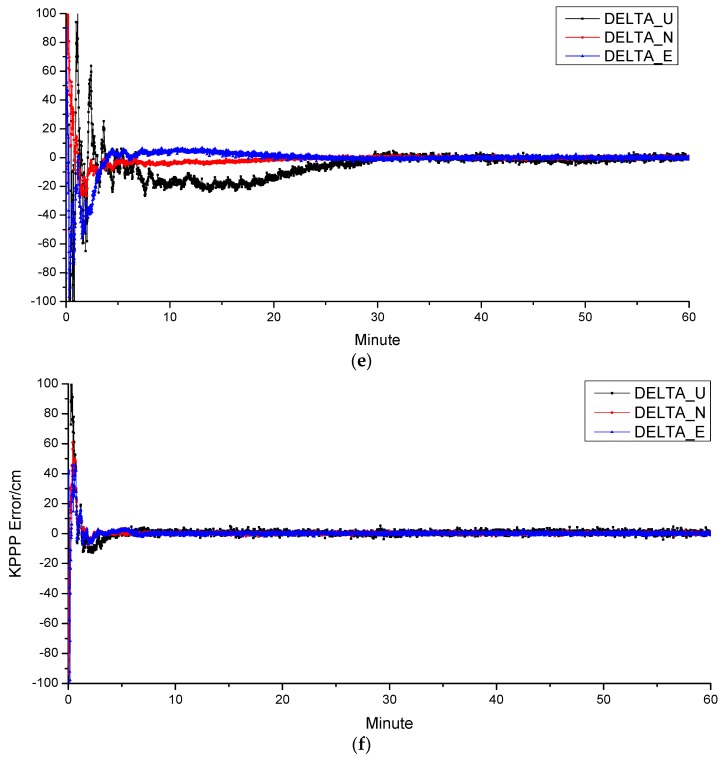
Time series of kinematic PPP positioning errors for BeiDou-3 and BeiDou-3 as well as LEO combined solutions. (**a**) CENT BeiDou-3 only; (**b**) CENT BeiDou-3 + LEO; (**c**) POTS BeiDou-3 only; (**d**) POTS BeiDou-3 + LEO; (**e**) NTUS BeiDou-3 only; (**f**) NTUS BeiDou-3 + LEO.

Figure 5 shows the positioning error in north, east, and up direction for the two solutions of the selected station. In general, the inclusion of LEO can significantly accelerate the positioning convergence. The convergence time can be shorted to 1 min 9 s from 28 min 35 s. The results indicates the most great advantage of LEO constellation, which can greatly accelerating the convergence speed of the ambiguities, and providing promising way for real-time PPP application.

**Figure 6:**
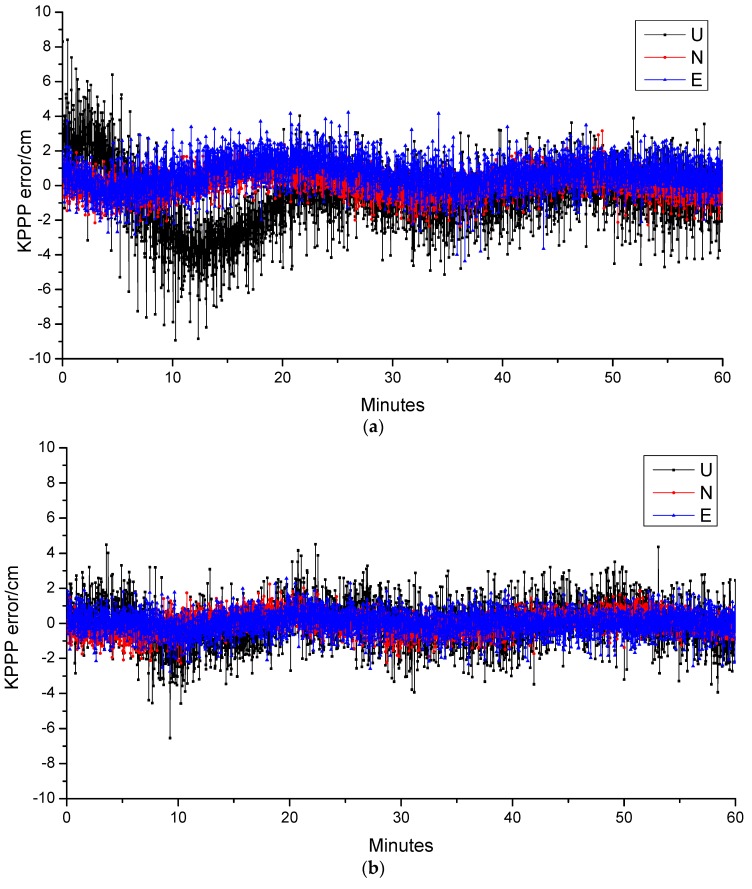
Time series of kinematic PPP positioning errors of CENT station in 1 h after the convergence of PPP for BeiDou-3 (**a**) and BeiDou-3 as well as LEO combined solutions (**b**), respectively.

Figure 6 further show the variations of kinematic positioning errors of CENT station in 1 h after the convergence of PPP for BeiDou-3 only and BeiDou-3 as well as LEO combined solutions. It is easy to notice that the positioning accuracy is within 5 cm in horizontal direction, whereas the it is within 8 cm in vertical direction. However, once the LEO data used, the variations of kinematic positioning errors become stable, and it is below 5 cm in all of the three direction. Statically, the RMS of position accuracy has been improved from 1.12, 0.74, and 2.05 cm to 0.65, 0.59, 1.23 cm in east, north, and up direction, once the LEO satellites are used to augment the BeiDou-3. The simulation results of all these three stations are listed in Table 8. This clearly demonstrate the advantage of LEO used as the navigation system.

## 5. Conclusions

Currently, the Global Navigation Satellite System (GNSS) mainly use the satellites in Medium Earth Orbit (MEO) to provide position, navigation, and timing (PNT) service. The weak navigation signals limit its usage in deep attenuation environments, and make it easy to interference and counterfeit by jammers or spoofers. Moreover, being far away to the Earth results in relative slow motion of the satellites in the sky and geometric change, making long time needed for achieved centimeter positioning accuracy. By using the satellites at lower earth orbit (LEO) as the navigation satellites, these disadvantages can be addressed. 

LEO satellites (with altitudes from 160 km to 2000 km) are 20 times closer to the Earth than GNSS at MEO, leading to stronger radio signals on the ground for navigation. The swift motion of LEO provides the rapid geometric change, which resulting quicker PDOP change and rapid convergence for integer ambiguity. We compared and analyze this theoretically. We have simulated the BeiDou-3 constellation consisting three GEO, three IGSO, and 24 MEO satellites and LEO navigation system consisting of a constellation of 120 satellites in 10 orbit planes with inclination of 55 degrees in a nearly circular orbit (eccentricity about 0.000001) at an approximate altitude of 975 km to assess the integrated navigation performance of LEO constellation. The results confirm the theoretical analysis, and demonstrate the satellite visibility and PDOP can be improved. More importantly, the accuracy and convergence of PPP can be improved and significantly reduced to about 1 min.

Although the simulation used in this study is used for augmenting BeiDou-3 with LEO constellation. The LEO constellation can also be used as independent navigation system, as Iridium does. However, as too close to the Earth, the ground converge of LEO satellites is less than one-tenth of that of MEO ones. Hence, hundreds of LEO satellites would be needed to match the coverage of GNSS. Thanks to the development of commercial communication LEO constellation, it is promising and possible to integrate the navigation components to the communication satellites to provide independent navigation from LEO. Currently, many plans have been proposed to achieve this around the world, such as Chinese Hongyun and Hongyan. Once it has been implemented, the real-time navigation with cm level accuracy will be achieved easily, and definitely significantly change our daily life.

## Figures and Tables

**Table 1:** The summary of some deployed or proposed commercial communication LEO constellations.

Constellation	No. Sats	Altitude [km]	Inclination [°]	Year	Country
Iridium	66	781	86.4	1998	USA
Globalstar	48	1400	52	2000	USA
LeoSat	108	1400	Not defined, yet	2019/2020	USA
Telesat	117	1000~1245	99.5	2021	Canada
Hongyun	156	1000	Not defined, yet	2022	China
Hongyan	324	1100	Not defined, yet	2023	China
OneWeb	648	1200	88	2019	USA/UK
Boeing	2956	1200	45, 55, 88	Not defined, yet	USA
SpaceX Starlink	7518	1110~1325	53, 53.8, 74, 81, 70	2020	USA
Astrome Technology	150	1400	Not defined, yet	2020	India
Samsung	4600	<1500	Not defined, yet	Not defined	Korea

**Table 2:** Ambiguity Solution Related to the Altitude of Satellite.

Altitude	Convergence Time for Ambiguity of Float Solution	Time for Integer Ambiguity Resolution
1000 km	1 min	10 min
10,000 km	7 min	1 h
20,000 km	20 min	4 h
IGSO 36,000 km	2 h	25 h
GEO 36,000 km	+∞	+∞

**Table 3:** Dynamic models used for generating the 24 h orbits for BeiDou-3 and LEO constellation.

Geophysical Models	Description	
	BeiDou-3	LEO
Static	EGM2008 up to degree and order 12	Static part of EIGEN-6C up to degree and order 150
Temporal	None	Temporal part of EIGEN-6C up to degree and order 50
Secular rates for low degree coefficients	IERS Conventions 2010 [25]	IERS Conventions 2010 [25]
n-body	Moon, Sun, Mercury, Venus, Mars, Jupiter, Saturn, Uranus, Neptune, Pluto JPL DE405	Moon, Sun, Mercury, Venus, Mars, Jupiter, Saturn, Uranus, Neptune, Pluto JPL DE405
Solid Earth Tides	IERS Conventions 2010 [25]	IERS Conventions 2010 [25]
Ocean Tides	None	FES2004
Ocean pole tides	None	Desai [26]
Relativistic effects	IERS Conventions 2010 [25]	IERS Conventions 2010 [25]
**Satellite surface models and Attitude**		
Atmospheric drag	None	DTM94 with Box-wing model
Solar Radiation Pressure	5-parameter ECOM model	Box-wing
Attitude	Nominal yaw-steering model	consistent with local orbital reference frame
**Reference frame**		
Inertial frame	J2000.0	J2000.0
Earth tide and Ocean loading	IERS Conventions 2010 [25]	IERS Conventions 2010 [25]
Precession/Nutation	IAU 2000A	IAU 2000A
EOP	IERS EOP 08 C04 (IAU2000A)	IERS EOP 08 C04 (IAU2000A)

**Table 4:** The configures for observation simulation.

Constellation	BeiDou-3	LEO
Cone angle	GEO/IGSO: 10	65
	MEO: 15	
PCO	(600, 0, 11,000) mm	(0, 0, 0)
PCV	0	0
Satellite clock	Clock file	Clock file
Mask elevation	5	5
PCO	0	0
PCV	0	0
Receiver clock	0	0
Solid/Ocean/Pole tide	IERS Conventions 2010	IERS Conventions 2010
Troposphere delay	Saastamonion for dray and wet delay	Saastamonion for dray and wet delay
	GMF	GMF
Ionosphere delay	No	No
Phase wide-up	Yes	Yes
Relativity	Yes	Yes
Code noise	1 m	1 m
Phase noise	2 mm	2 mm
Ambiguity	0	0

**Table 5:** Statistical results for satellite visibility.

Constellation	Max. # of Tracked Satellites	Min. # of Tracked Satellites	Avg. # of Tracked Satellites
3GEO + 3IGSO + 24MEO	15.5	7.6	10.7
3GEO + 3IGSO + 24MEO + 120LEO	21.6	10.1	16.3

**Table 6:** Statistical results for PDOP.

Constellation	Max. PDOP	Min. PDOP	Avg. of PDOP
3GEO + 3IGSO + 24MEO	2.24	1.19	1.63
3GEO + 3IGSO + 24MEO + 120LEO	1.93	1.01	1.22

**Table 7:** SPP precision of both BDS and BDS + LEO (cm).

Station	BDS			BDS + LEO		
	North	East	Up	North	East	Up
CENT	99.1	105.3	293.7	93.2	100.7	261.3
POTS	152.7	131.8	290.7	121.3	87.4	229.9
NTUS	91.6	114.9	217.2	84.5	101.5	199.2

**Table 8:** PPP precision of both BDS and BDS + LEO after the ambiguity convergence (cm).

Station	BDS			BDS + LEO		
	North	East	Up	North	East	Up
CENT	0.74	1.12	2.05	0.59	0.65	1.23
POTS	0.93	0.99	1.96	0.50	0.72	1.44
NTUS	0.54	0.68	1.33	0.63	0.49	1.23
